# Clinical outcomes of early gastric cancer with non-curative resection after pathological evaluation based on the expanded criteria

**DOI:** 10.1371/journal.pone.0224614

**Published:** 2019-10-31

**Authors:** Hyun Ju Kim, Sang Gyun Kim, Jung Kim, Hyoungju Hong, Hee Jong Lee, Min Seong Kim, Hyunsoo Chung, Hyun Chae Jung

**Affiliations:** 1 Department of Internal Medicine and Liver Research Institute, Seoul National University College of Medicine, Seoul, Korea; 2 Yonsei University Graduate School of Medicine, Seoul, Korea; 3 Health Promotion Center, Seoul National University Hospital, Seoul, Korea; Tata Memorial Centre, INDIA

## Abstract

Additional surgical resection should be considered for the patients with pathological findings beyond the expanded criteria with the risk for LN metastasis. However, close observation without additional surgery may be applied because of various reasons. We aimed to determine the clinical outcomes of early gastric cancer beyond the expanded criteria after endoscopic resection according to the pathological extent. A total of 288 patients with 289 lesions beyond the expanded criteria of endoscopic submucosal dissection for early gastric cancer were analyzed between 2005 and 2016, and classified into two groups according to additional treatment: observation (n = 175 patients, 175 lesions) and surgery (n = 113 patients, 114 lesions). The depth of tumor invasion was greater and the tumor-positive vertical margin and lymphatic and venous invasion were more common in the surgery group than in the observation group (P<0.001). Residual, synchronous, and metachronous tumors were more common in the observation group; however, the occurrence of regional lymph node and distant metastasis did not differ between the groups. Overall survival and 5-year disease-specific survival did not differ between the groups (observation vs surgery, 88.6 vs 93.8%; P = 0.259, 98.2 vs 100%; P = 0.484, respectively), but the 5-year disease-free survival was lower in the observation group (73.5 vs 97.9%; P<0.001). On multivariate analysis, tumor-positive lateral margin was a risk factor for residual tumor and lymphatic and venous invasion were risk factors for regional lymph node metastasis. In conclusion, the clinical course of beyond the expanded criteria of endoscopic submucosal dissection for early gastric cancer showed good prognosis over 98% in 5-year disease specific survival. If additional surgery cannot be performed, a close follow-up with endoscopy and abdominal computed tomography can be considered as an alternative for carefully selected patients without lymphatic and vascular invasion.

## Introduction

Gastric cancer is one of the predominant cancers and is the third major cause of cancer-related deaths. The early detection of gastric cancer has increased in Korea because of the national cancer screening program [1]. Endoscopic submucosal dissection (ESD) is currently a preferred curative treatment modality for early gastric cancer (EGC) and premalignant gastrointestinal epithelial lesions without the risk for lymph node (LN) metastasis [2].

The expanded criteria for the endoscopic resection of EGC with a negligible risk of LN metastasis in the review of surgical cases were proposed as follows: (1) differentiated intramucosal adenocarcinoma without ulceration, regardless of the tumor size; (2) differentiated intramucosal adenocarcinoma, smaller than 3 cm in diameter, with ulceration; (3) differentiated submucosal superficial adenocarcinoma (< 500 μm from the muscularis mucosa), smaller than 3 cm; and (4) undifferentiated intramucosal cancer, smaller than 2 cm, without ulceration [3]. The Japan Gastroenterological Endoscopy Society (JGES) guideline defines the curative expanded criteria as en bloc resection with a negative tumor margin and negative lymphatic and vascular invasion, and recommended additional surgery for patients who do not meet the curative expanded criteria [4].

Regardless of the en bloc status, surgical margin, and lymphatic and vascular invasion, additional surgical resection should be considered for the patients with pathological findings beyond the expanded criteria with the risk for LN metastasis. However, close observation without additional surgery may be applied because of underlying serious comorbidities, old age, patients’ wish, or other reasons.

Some studies have investigated the clinical outcomes of ESD including beyond-the-expanded criteria[5–8] and clinical outcomes and additional treatment after non-curative ESD for EGC[9–13], but there has been only one study of different outcomes of ESD according to the additional treatment, with a focus on lesions beyond the expanded criteria of ESD for EGC, rather than on non-curative ESD for EGC [8].

Therefore, we investigated the clinical outcomes and the risk of LN metastasis of patients with pathological findings beyond the expanded criteria, regardless of the en bloc status, surgical margin, and lymphatic and vascular invasion, according to additional surgical resection.

## Materials and methods

### Patients

Of the 2453 patients diagnosed with EGC who underwent ESD between January 2005 and December 2016 at Seoul National University Hospital, Seoul, Korea, 303 (12.4%) who were classified as having EGC beyond the expanded criteria were retrospectively analyzed. Lesions which did not meet the expanded criteria following pathological evaluation were classified as being “beyond the expanded criteria”, regardless of the en bloc status, surgical margin, and lymphatic and vascular invasion [4]. Patients who were lost to follow-up within 6 months after ESD were excluded from the analysis. The patients were classified into two groups according to additional treatment: observation and surgery groups.

The study design was approved by the Institutional Review Board of Seoul National University Hospital (H-1704-123-848). The study was exempted from the requirement to obtain informed consent.

### ESD procedure and histologic examination

A standard single-channel endoscope (Olympus H260, Olympus Optical) as described previously was used for ESD [14]. Briefly, after marking several points at 5 mm around the lesion using a forced 20-W coagulation current (VIO 300D; Erbe, Tübingen, Germany) needle-knife (KD- 1L; Olympus), a solution containing mixture of normal saline, diluted epinephrine (1:100,000), and indigo carmine was injected to lift the submucosal layer. After that, a small initial incision was made with a needle-knife. Thereafter, a circumferential mucosal incision and submucosal layer dissection were made using an insulation-tipped knife (Kachu Technology Co. Ltd., Seoul, Korea). Japanese Classification of Gastric Carcinoma (JCGC) [15] was used to determine the location and macroscopic type of the EGC lesions. Type 0-I (protruding) and 0-IIa (superficial elevated) were grouped together as the elevated type, whereas, type 0-IIb (superficial flat), 0-IIc (superficial depressed), 0-III (excavated), 0-IIa+IIc, and 0-IIb+IIc were grouped together as the non-elevated type. The resected specimens were promptly stretched and pinned on a flat polystyrene board to prevent folding and fixed in 10% formalin. For histologic evaluation, the fixed specimens were serially sectioned at 2-mm intervals and the histologic type, tumor size, invasion depth, tumor involvement, and lymphovascular invasion were assessed according to the JCGC.

### Follow-up

Chest radiography was performed after endoscopic resection to ascertain the presence of perforations. Proton pump inhibitor was administered intravenously from the day of the procedure and then orally for 6 weeks to heal artificial ulcers. Patients were discharged the next day if there was absence of bleeding or perforation. Second-look endoscopy was not usually performed.

Patients with findings beyond the expanded criteria in the final pathologic evaluation were recommended for additional surgical resection. However, a close observation approach without surgical resection was also adopted for some elderly patients (>80 years), those with severe comorbidities, or upon patients' refusal. Follow-up surveillance endoscopy was performed at 3, 6, and 12 months, and annually thereafter, and abdominal computed tomography and chest radiography were performed at 6 and 12 months, and annually thereafter to detect tumor recurrence.

### Clinical outcomes

Clinical outcomes were evaluated in terms of tumor recurrence, survival, and the risk factors for residual tumor or LN metastasis.

Residual tumor was defined as remnant tumor at the ESD scar at follow-up endoscopy, synchronous tumor as new cancer development elsewhere other than at the ESD scar in the stomach within 12 months after the first ESD, and metachronous tumor as tumor development beyond 12 months after the first ESD.

Overall survival (OS) was defined as the time from the date of diagnosis until the date of death. Disease-specific survival (DSS) was calculated from the date of diagnosis to the date of death related to gastric cancer. Disease-free survival (DFS) was measured from the time of ESD to initial tumor relapse or death.

### Data analysis

The following demographic and clinical parameters, namely patient-related factors, tumor-related factors, and clinical outcomes, were compared based on additional treatment modality.

The t-test, χ^2^ test, and Fisher’s exact test were used for comparisons between two independent groups. Survival analysis was performed using the Kaplan-Meier method and log-rank test. Patient- and tumor-related factors were included as potential risk factors for residual tumor, regional LN metastasis, distant metastasis, and death in the univariate analyses. Risk factors with a *P*-value of <0.05 in the univariate analyses were entered into the multiple logistic regression model and analyzed using the stepwise forward-selection method. Odds ratios (ORs) and 95% confidence intervals (CIs) were calculated for risk factors. The 95% CI of the OR was used to assess statistical significance at *P*<0.05. SPSS for Windows version 20.0 (SPSS Inc., Chicago, IL) was used for all statistical analyses.

## Results

### Patient and lesion characteristics

**Fig 1:**
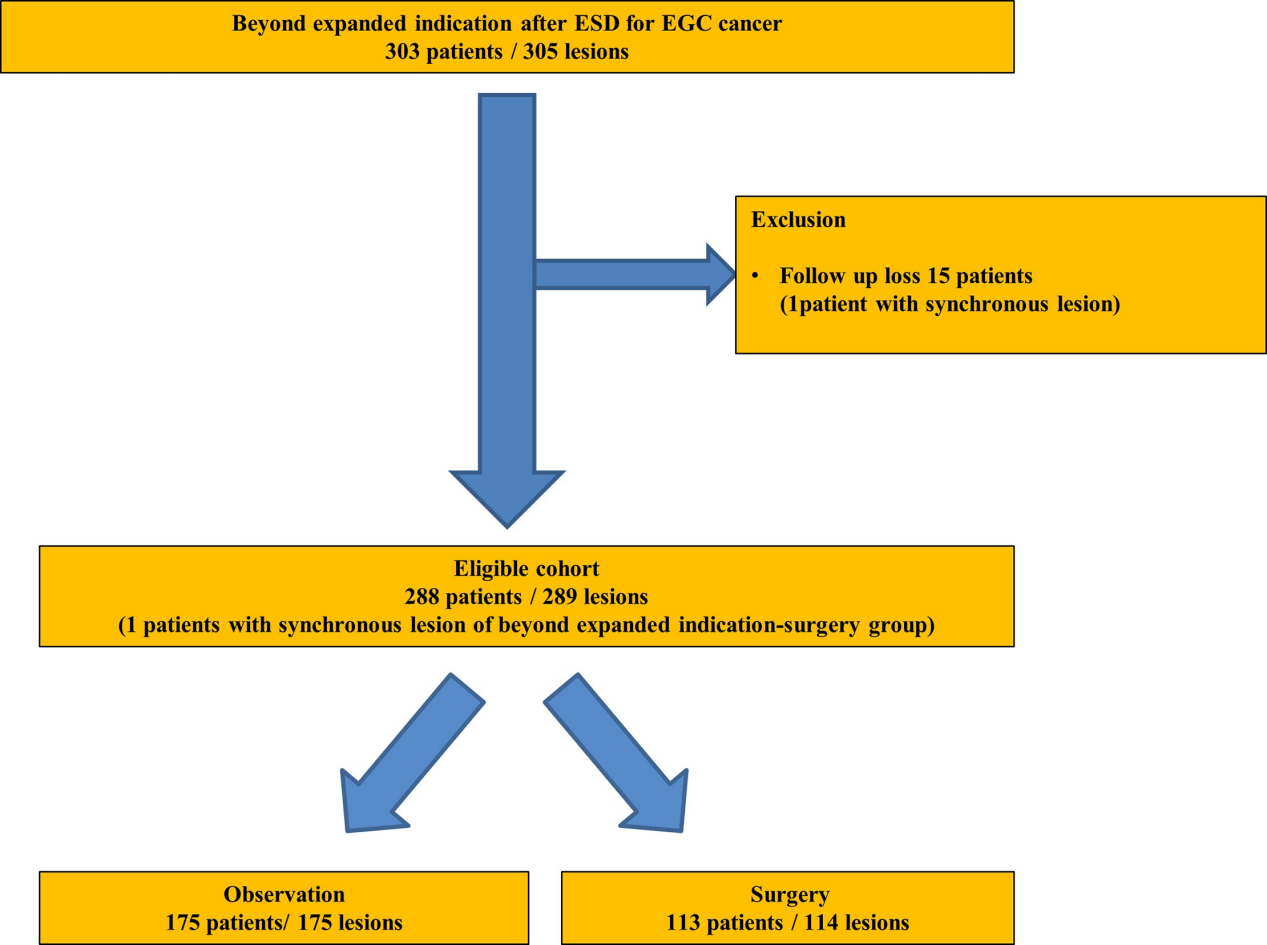
Study flow diagram. ESD, endoscopic submucosal dissection; EGC, early gastric cancer.

Among 288 patients with 289 lesions beyond the expanded criteria, 175 patients (60.8%) with 175 lesions (60.6%) were categorized to the observation group and 113 patients (39.2%) with 114 lesions (39.4%) to the surgery group (Fig 1). Regarding patient characteristics, all factors, including age, sex, and underlying disease, did not differ significantly between both groups (Table 1). Macroscopically flat or depressed-type lesions and undifferentiated histology were more common in the observation group than in the surgery group (88.6 vs 78.1%; *P* = 0.016, 52.0 vs 28.9%; *P*<0.001, respectively), and the mean tumor size was larger in the observation group than in the surgery group (2.9 ± 1.3 vs 2.5 ± 1.3 mm; *P* = 0.022). However, the depth of tumor invasion was less and the tumor-positive vertical margin and lymphatic and venous invasion were less common in the observation group than in the surgery group (0.9 ± 0.6 vs. 1.3 ± 0.8 mm; *P*<0.001, 8.0 vs. 60.5%; *P*<0.001, 7.4 vs. 43.9%; *P*<0.001, 0.6 vs. 18.4%; *P*<0.001, respectively). The other characteristics did not significantly differ between the two groups (Table 2).

**Table 1:** Patient characteristics.

Variable	Total	Observation	Surgery	*P* value
**Patient, no**	288	175	113	
**Age, y, mean ± SD**	63.5 ± 0.6	63.9 ± 10.6	62.7 ± 9.2	0.324
**Male, no. (%)**	192 (65.3)	112 (64.0)	80 (70.8)	0.232
**Underlying disease, no. (%)**				
**Hypertension**	112 (38.1)	67 (38.3)	45 (40.2)	0.748
**Diabetes**	62 (21.1)	34 (19.4)	28 (24.8)	0.281
**Cardiovascular disease**	25 (8.5)	15 (8.6)	10 (8.8)	0.935
**Chronic kidney disease**	3 (1.0)	2 (1.1)	1 (0.9)	>0.999
**Cerebrovascular event**	11 (3.7)	8 (4.6)	3 (2.7)	0.538
**Liver cirrhosis**	19 (6.5)	10 (5.8)	9 (8.0)	0.456
**Alcohol, no. (%)**				0.306
**No**	204 (69.4)	121 (69.1)	83 (73.5)	
**Yes**	83 (28.2)	54 (30.9)	29 (25.7)	
**Unknown**	1 (0.3)	0 (0)	1 (0.9)	
**Smoking, no. (%)**				0.799
**No**	229 (77.9)	140 (80.0)	89 (78.8)	
**Yes**	59 (20.1)	35 (20.0)	24 (21.2)	
**H.pylori, no. (%)**				0.686
**No**	124 (42.2)	75 (42.9)	49 (43.4)	
**Yes**	143 (48.6)	89 (50.9)	54 (47.8)	
**Unknown**	21 (7.1)	11 (6.3)	10 (8.8)	
**Previous ESD, no. (%)**				0.806
**No**	261 (88.8)	158 (90.3)	103 (91.2)	
**Yes**	27 (9.2)	17 (9.7)	10 (8.8)	
**Number of lesion, no. (%)**				0.770
**1**	276 (93.9)	167 (95.4)	109 (96.5)	
**≥2**	12 (4.1)	8 (4.6)	4 (3.5)	
**Reasons for observation**				
**Pt. refuse surgery**		59 (33.7)		
**Old age**		37 (21.1) 9(		
**Poor general condition**		16 (9.1)		
**Other cancer**		10 (5.7)		
**Physician’s decision**		53 (30.3)		

SD, standard deviation; ESD, endoscopic submucosal dissection; Pt, patient

**Table 2:** Lesion characteristics of 1^st^ ESD.

Variable	Total	Observation	Surgery	*P* value
**Lesions, no**	289	175	114	
**Location, no. (%)**				0.558
**Lower third**	242 (83.7)	144 (82.3)	98 (86.0)	
**Middle third**	18 (6.2)	13 (7.4)	5 (4.4)	
**Upper third**	29 (10.0)	18 (10.3)	11 (9.6)	
				0.150
**AW**	67 (23.2)	38 (21.8)	29 (25.4)	
**LC**	88 (30.4)	55 (31.6)	33 (28.9)	
**PW**	130 (45.0)	81 (46.6)	49 (43.0)	
**GC**	3 (1.0)	0 (0.0)	3 (2.6)	
**Macroscopic appearance, no. (%)**				0.016
**Elevated type**	45 (15.6)	20 (11.4)	25 (21.9)	
**Non-elevated type**	244 (84.4)	155 (88.6)	89 (78.1)	
**Presence of ulcer, no. (%)**	50 (17.3)	30 (17.1)	20 (17.5)	0.930
**En bloc, no. (%)**	288 (99.7)	174 (99.4)	114 (100)	>0.999
**Differentiation of surgical pathology, no. (%)**				<0.001
**Differentiated**	165 (57.1)	84 (48.0)	81 (71.1)	
**Undifferentiated**	124 (42.9)	91 (52.0)	33 (28.9)	
**Lesion size on surgical pathology, mm,** **mean ± SD**	2.7 ± 0.1	2.9 ± 1.3	2.5 ± 1.3	0.022
**Depth of invasion on surgical pathology, no. (%)**				<0.001
**pT1a**	91 (31.5)	86 (49.1)	5 (4.4)	
**pT1b**	198 (68.5)	89 (50.9)	109 (95.6)	
				<0.001
**Sm1**	26 (9.0)	18 (20.2)	7 (6.4)	
**Sm2**	135 (46.7)	64 (71.9)	72 (66.1)	
**Sm3**	37 (12.8)	7 (7.9)	30 (27.5)	
**≥0.5mm**	173 (59.9)	71 (40.6)	102 (89.5)	<0.001
**Depth of invasion on surgical pathology, mm, mean ± SD**	1.1 ± 0.1	0.9 ± 0.6	1.3 ± 0.8	<0.001
**Beyond expanded criteria**				<0.001
**Only beyond expanded criteria**^**a**^	130 (45.0)	120 (68.6)	10 (8.8)	
**With the other conditions**^**b**^	159 (55.0)	55 (31.4)	104 (91.2)	
**Lateral margin (+), no (%)**	51 (17.6)	33 (18.9)	18 (15.8)	0.504
**Vertical margin (+), no (%)**	83 (28.7)	14 (8.0)	69 (60.5)	<0.001
**Lymphatic invasion (+), no (%)**	63 (21.8)	13 (7.4)	50 (43.9)	<0.001
**Venous invasion (+), no (%)**	22 (7.6)	1 (0.6)	21 (18.4)	<0.001
**Perineural invasion (+), no (%)**	4 (1.4)	1 (0.6)	3 (2.6)	0.304

ESD, endoscopic submucosal dissection; AW, anterior wall; LC, lesser curvature; PW, posterior wall; GC, greater curvature; SD, standard deviation

^a^Only beyond expanded Criteria: beyond expanded criteria with En bloc resection, negative tumor margin, negative lymphatic and vascular invasion

^b^With the other conditions: beyond expanded criteria with piecemeal resection or positive tumor margin or positive lymphatic invasion or positive vascular invasion

### Final pathologic outcomes of the surgery group

Among patients who underwent additional surgical resection, residual tumor was found in 20.4%. In the case of residual tumor, pT1a and pT1b were 8.8% and 6.1%, respectively, and pT2 and pT3 were also noted to be 4.4% and 0.9%, respectively. Regional LN metastasis was found in 13.2% and incidental synchronous lesion, including adenoma, cancer, and neuroendocrine tumor, was found in 5.3% (Table 3).

**Table 3:** Final pathology result of surgery group.

Variable	N (%)
**Patient, no**	113
**Differentiation of surgical pathology, no. (%)**	
**No residual tumor**	90 (79.6)
**Differentiated**	15 (13.3)
**Undifferentiated**	8 (7.1)
**Depth of invasion on surgical pathology, no. (%)**	
**pT1a**	10 (8.8)
**pT1b**	7 (6.1)
**pT2**	5 (4.4)
**pT3**	1(0.9)
**≥0.5mm**	10 (8.8)
**pN stage, no (%)**	
**pN0**	98 (86.7)
**pN1**	11 (9.7)
**pN2**	4 (3.5)
**Incidental synchronous lesion**	6 (5.3)
**Adenoma**	1(0.9)
**Cancer**	4 (3.5)
**Neuroendocrine tumor**	1(0.9)

### Tumor recurrence during follow-up

In the observation and surgery groups, the median follow-up periods were 55.6 and 58.4 months, respectively (*P* = 0.162). Residual tumor, synchronous tumor, and metachronous tumor were more common in the observation group (9.7 vs. 0%; *P*<0.001, 5.1 vs. 0%; *P* = 0.013, 9.7 vs. 0.9%; *P* = 0.003, respectively), but regional LN metastasis and distant metastasis and overall and gastric cancer-related death did not differ between the groups.

Considering surgical pathology, there was no difference in synchronous tumor, distant metastasis, and overall and gastric cancer-related death between the groups. Although metachronous tumor was more common in the observation group, residual tumor and regional LN metastasis were more common in the surgery group (Table 4).

**Table 4:** Clinical outcomes of the gastric cancers after 1^st^ ESD according to additional treatment.

Variable	After additional treatment				Including surgical pathology of surgery group			
	Total	Observation	Surgery	*P* value	Total	Observation	Surgery	*P* value
**Patient, no**	288	175	113			175	113	
**Follow-up period, mo, median (range)**	55.6 (6–132)	55.6 (6–132)	58.4 (6–117)	0.162	55.6 (6–132)	55.6 (6–132)	58.4 (6–117)	0.162
**Residual tumor, no. (%)**	17 (5.9)	17 (9.7)	0 (0)	0.001	40 (13.9)	17 (9.7)	23 (20.4)	0.011
**Duration for recurrence, mo, median (range)**	11.8 (2.1–57.3)	11.8 (2.1–57.3)	N/A	N/A				
**Residual tumor depth ≥ T2** **, no (%)**	1 (0.3)	1 (0.6)	0 (0)	>0.999	7 (2.4)	1 (0.6)	6 (5.3)	0.016
**Synchronous tumor, no. (%)**	9 (3.1)	9 (5.1)	0 (0)	0.013	14 (4.9)	9 (5.1)	5 (4.4)	0.782
**Duration for recurrence, mo, median (range)**	6.1 (3.0–11.9)	6.1 (3.0–11.9)	N/A	N/A				
**Metachronous tumor, no. (%)**	18 (6.3)	17 (9.7)	1 (0.9)	0.003	18 (6.3)	17 (9.7)	1 (0.9)	0.003
**Duration for recurrence, mo, median (range)**	32.1 (12.0–126.5)	32.5 (12.0–126.5)	14.1 (14.1–14.1)	0.333				
**Regional LN metastasis, no. (%)**	3 (1.0)	3 (1.7)	0 (0)	0.282	18 (6.3)	3 (1.7)	15 (13.3)	<0.001
**Duration for recurrence, mo, median (range)**	45.2 (28.0–77.9)	45.2 (28.0–77.9)	N/A	N/A				
**Distant metastasis, no. (%)**	4 (1.4)	3 (1.7)	1 (0.9)	>0.999	4 (1.4)	3 (1.7)	1 (0.9)	>0.999
**Duration for recurrence, mo, median (range)**	36.6 (14.2–77.9)	45.26 (28.0–77.9)	14.2 (14.2–14.2)	0.500				
**Death, no. (%)**	34 (11.8)	23 (13.1)	11 (9.7)	0.381	34 (11.8)	23 (13.1)	11 (9.7)	0.381
**Gastric cancer- related death, no. (%)**	4 (1.4)	3 (1.7)	1 (0.9)	>0.999	4 (1.4)	3 (1.7)	1 (0.9)	>0.999

ESD, endoscopic submucosal dissection

In 17 residual tumors in the observation group, 2 lesions were observed and 10, 3, and 2 lesions were managed with argon plasma coagulation (APC), ESD, and surgery, respectively. The 2 patients who were observed without treatment died from ampulla-of-Vater cancer and hepatocellular carcinoma, respectively. One patient who was treated with APC for residual tumor underwent a total of 3 APC sessions. However, another metachronous tumor had developed and the patient died from gastric cancer regional and distant metastasis because the patient refused additional treatment.

In 9 patients with synchronous tumor in the observation group, 1 patient was observed, 1 tumor was removed by biopsy forceps alone, 2 tumors were ablated with APC, and 5 tumors were removed by ESD. All cases with APC or ESD were curatively treated and no one died from gastric cancer.

In 17 patients with metachronous tumor in the observation group, 1 patient was observed, 2 tumors were ablated with APC, 8 tumors were removed by ESD, and 4 tumors were removed by surgery. Among the patients, 1 patient underwent adjuvant chemotherapy after surgery and 1 patient refused additional treatment; all died of gastric cancer with regional LN metastasis and distant metastasis. One patient with metachronous tumor in the surgery group underwent curative APC.

Three patients died of gastric cancer in the observation group. One patient had a histological finding of a 2.8 cm-sized mucosa-confined poorly differentiated adenocarcinoma in the first ESD and underwent surgery and adjuvant chemotherapy for metachronous tumor in stage pT3N0. However, the patient died of gastric cancer with regional LN and distant metastasis. The second had a 4.3 cm-sized, submucosa-invasive, tumor-positive lateral margin, well-differentiated adenocarcinoma with lymphatic invasion in the first ESD and died of gastric cancer with regional LN and distant metastasis after refusing additional treatment for metachronous tumor. The third patient had a 1.2 cm-sized, submucosa-invasive, tumor-positive vertical-margin, moderately differentiated adenocarcinoma in the first ESD and died of gastric cancer, with regional LN metastasis in 14.8 months and distant metastasis in 28 months.

Only 1 patient died of gastric cancer in the surgery group. This patient had a 4.6 cm-sized, submucosa-invasive, tumor-positive vertical-margin, moderately differentiated adenocarcinoma with lymphatic and venous invasion in the first ESD, with a final surgical stage of T1bN2. The patient died of gastric cancer with distant metastasis after adjuvant chemotherapy.

**Fig 2:**
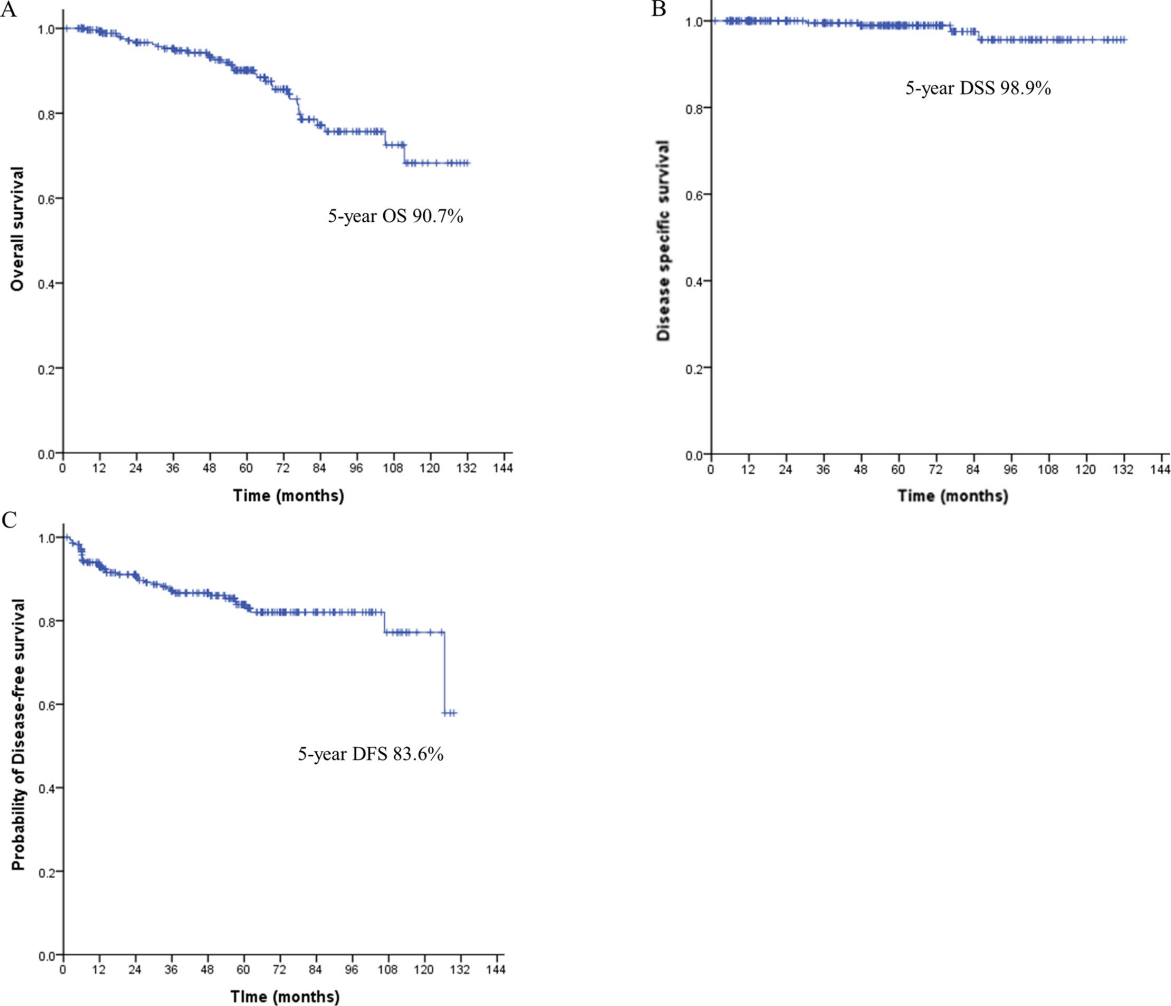
Kaplan-Meier plots for overall survival (A), disease-specific survival (B), and disease-free survival (C) in all the patients. OS, overall survival; DSS, disease-specific survival; DFS, disease-free survival.

**Fig 3:**
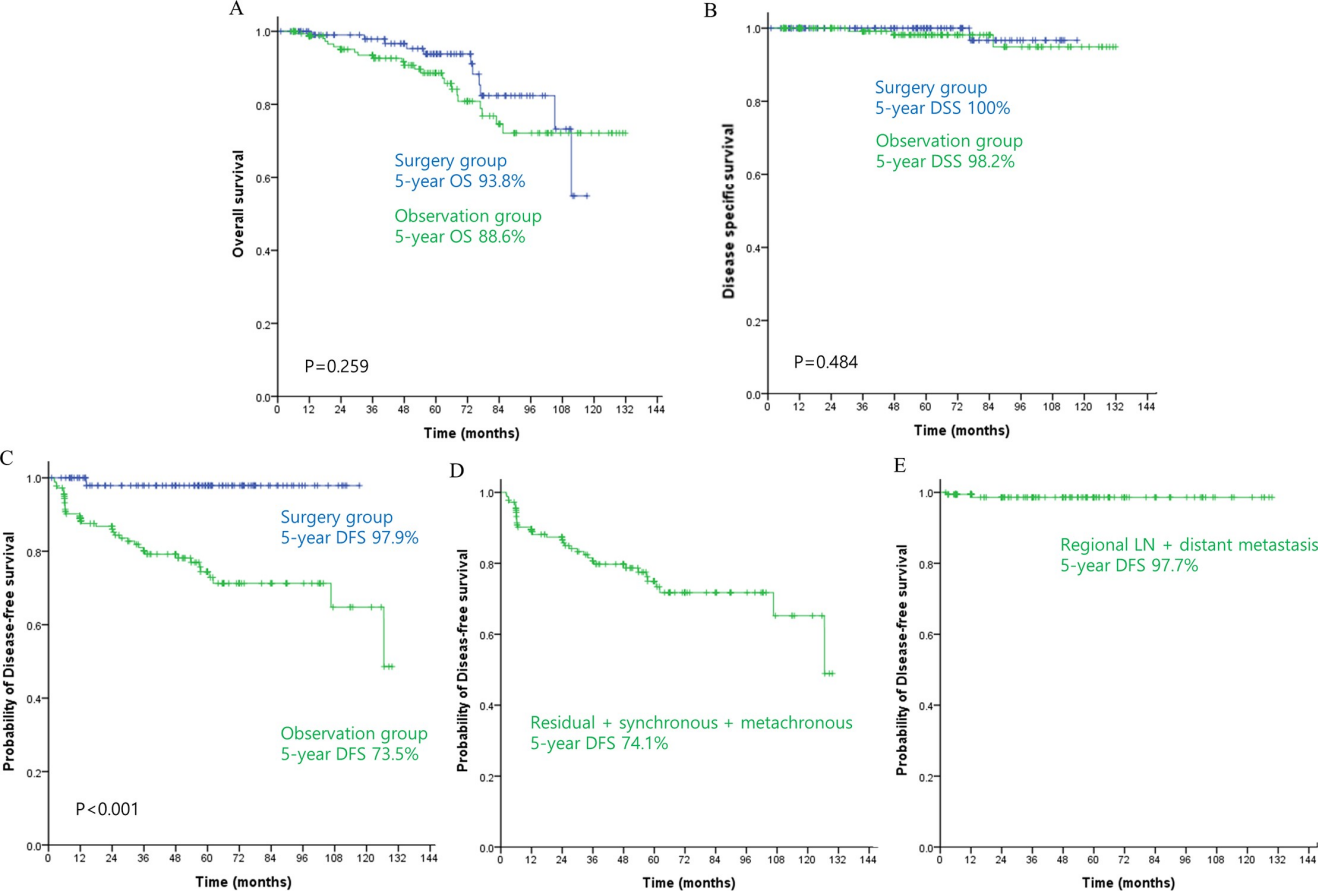
Kaplan-Meier plots for overall survival (A), disease-specific survival (B), and disease-free survival (C) according to treatment type: observation group (green line) versus surgery group (blue line). Disease-free survival (residual + synchronous + metachronous tumor) (D) and disease-free survival (regional LN + distant metastasis) (E) in the observation group. OS, overall survival; DSS, disease-specific survival; DFS, disease-free survival; LN, lymph node.

Five-year OS was 90.7%, 5-year DSS 98.9%, and 5-year DFS 83.6% (Fig 2). The 5-year OS and 5-year DSS did not differ between the groups (observation vs surgery; 88.6 vs 93.8%; *P* = 0.259 and 98.2 vs 100%; *P* = 0.484), but 5-year DFS was lower in the observation group (73.5 vs 97.9%; *P*<0.001). In the observation group, residual, synchronous, and metachronous tumor were more common than regional LN and distant metastasis (74.1% vs 97.7%) (Fig 3).

**Fig 4:**
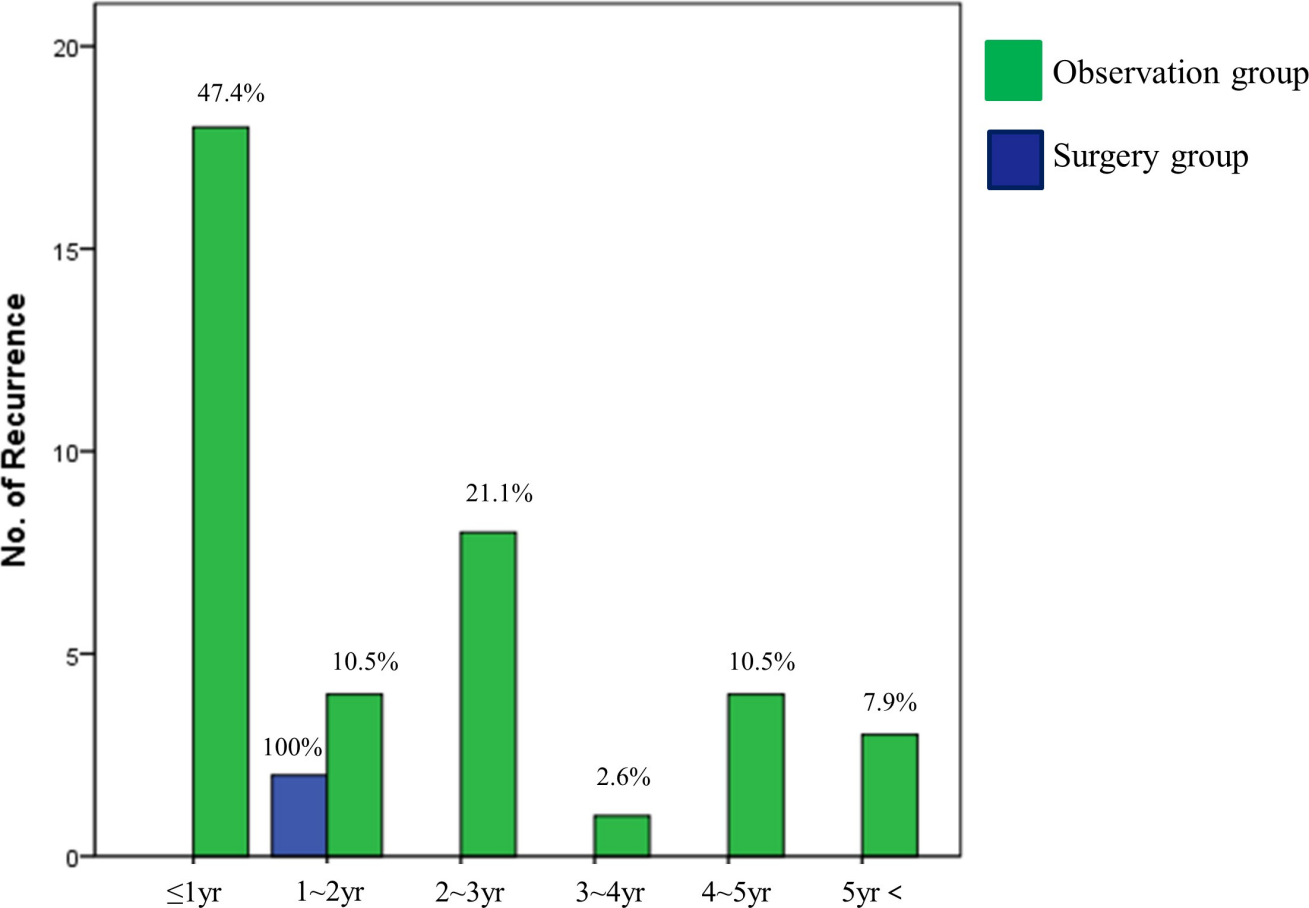
Comparison of time to recurrence between the two groups. (P = 0.036) observation group (green) versus surgery group (blue).

In the analysis, according to the time to recurrence, the tumor recurrence rate was 100% between 1 and 2 years in the surgery group and 47.4% within the first year, and 7.9% after 5 years in the observation group; a significant difference was noted between both groups (*P* = 0.036) (Fig 4).

### Risk factors for residual tumor, synchronous tumor, and regional LN metastasis

On multivariate analysis, a tumor-positive lateral margin was a risk factor for residual tumor and tumor multiplicity was a risk factor for synchronous tumor (OR: 19.92, 95% CI: 8.71–45.53, *P*<0.001; OR: 8.03, 95% CI: 1.90–33.86, *P* = 0.005, respectively). Lymphatic and venous invasion were risk factors for regional LN metastasis (OR: 4.01, 95% CI: 1.36–11.81, *P* = 0.012; OR: 5.85, 95% CI: 1.83–18.67, *P* = 0.003, respectively) (Table 5).

**Table 5:** Risk factors for residual tumor, residual tumor with depth ≥T2, synchronous tumor and regional LN metastasis of beyond expanded criteria of ESD for EGC.

Factor	Univariate analysis	Multivariate analysis	
	*P* value	OR (95% CI)	*P* value
**Residual tumor**			
**Lesion size (mm)**	0.001	1.20 (0.87–1.66)	0.254
**Lateral margin (negative vs positive)**	<0.001	19.92 (8.71–45.53)	<0.001
**Residual tumor with depth ≥T2**			
**Presence of ulcer (no vs yes)**	0.019	10.41 (1.65–65.77)	0.013
**Lesion size (mm)**	0.013	2.00 (1.04–3.86)	0.037
**Lateral margin (negative vs positive)**	0.020	8.46 (1.30–55.14)	0.025
**Vertical margin (negative vs positive)**	0.022	15.74 (2.19–113.03)	0.006
**Synchronous tumor**			
**Number of lesion (1 vs ≥2)**	0.016	8.03 (1.90–33.86)	0.005
**Regional LN metastasis**			
**Depth of invasion (pT1a vs pT1b)**	0.011	1.64 (0.08–32.62)	0.743
**Depth of invasion (<0.5 vs ≥0.5mm)**	0.006	1.95 (0.19–19.32)	0.568
**Vertical margin (negative vs positive)**	0.004	1.65 (0.53–5.13)	0.383
**Lymphatic invasion (negative vs positive)**	<0.001	4.01 (1.36–11.81)	0.012
**Venous invasion (negative vs positive)**	<0.001	5.85 (1.83–18.67)	0.003

LN, lymph node; ESD, endoscopic submucosal dissection; EGC, early gastric cancer; OR, odds ratio

Considering these risk factors, we performed prognostic grouping according to the following criteria: group 1 (*n* = 218) without any risk factor, group 2 (*n* = 60) with lymphatic invasion or venous invasion, and group 3 (*n* = 11) with lymphatic and venous invasion. Regional LN metastasis occurred in 2.8% of group 1, 13.8% of group 2, and 45.5% of group 3, and OR for regional LN metastasis, with group 1 as the reference group, was 5.43, and 29.44 for group 2, and 3, respectively (Table 6).

**Table 6:** Regional LN metastasis risk according to risk group as defined by the prognostic model.

Risk group	Risk factors	No. of lesions (%)	LN metastasis (%)	OR (95% CI) for regional LN metastasis
**Group 1**	No	218 (75.4)	6 (2.8)	1
**Group 2**	Lymphatic or venous invasion	60 (20.8)	8 (13.3)	5.43 (1.80–16.34)
**Group 3**	Lymphatic & Venous invasion	11 (3.8)	5 (45.5)	29.44 (6.99–123.98)

LN, lymph node; OR, odds ratio

## Discussion

Although additional surgery is indispensable for histology-identified lesions beyond the expanded criteria of ESD for EGC regardless of the en bloc status, surgical margin, and lymphatic and vascular invasion, only 40–67.6% of patients underwent additional treatment in previous studies [5–8]. Similar to previous studies, additional surgery was performed only in 39.2% of the patients in this study because of old age, poor general condition, other-cancer status, patients’ refusal, or some physicians’ decisions. In this study, we confirmed once again that the risk factors of lymph node metastasis were lymphatic and venous invasion and only focused on beyond the expanded criteria of endoscopic submucosal dissection for early gastric cancer which is strongly required for surgery, and that the clinical course of beyond the expanded criteria of endoscopic submucosal dissection for early gastric cancer without the two risk factors was not very hopeless. It will be more helpful for the GI endoscopists to inform the patients regarding the prognosis of beyond the expanded criteria of endoscopic submucosal dissection for early gastric cancer, and thus help determine between observation and additional surgery.

In previous studies on the clinical outcome of lesions beyond the expanded criteria and within the absolute and expanded criteria of ESD for EGC, the 5-year OS of beyond the expanded criteria was 84.4–94.1%, which was lower or not different compared to the expanded group [5, 16]. In beyond the expanded criteria, disease-specific death was 3.9% and the 5-year DSS rate was 97.4%, which did not differ between the absolute, expanded, and beyond the expanded criteria (*P* = 0.088).[5, 6] In this study, the 5-year OS and the 5-year DSS in the beyond the expanded criteria group were slightly lower than the two previous studies including the expanded criteria (5-year OS: 96.2–97.1%, 5-year disease-specific OS: 100%) [17, 18].

Residual, synchronous, and metachronous tumor occurred more frequently in the observation group than in the surgical group, which were mostly cured by APC, ESD, or additional surgery. As all the recurrence occurred between 1 and 2 years after ESD in the surgery group, frequent and careful follow-up may be mandatory at an early stage in this group. However, recurrence occurred in 47.4% of patients within the first year and in 7.9% after 5 years in the observation group. Thus, careful follow-up may be required frequently at an early stage and in the long term in this group.

Unlike previous studies in which old age, undifferentiated histology, and tumor size were mentioned as risk factors for synchronous tumors [19–21], the risk for synchronous tumors was tumor multiplicity at the time of the first ESD in this study. Patients with multiple tumors beyond the expanded criteria at the time of first ESD may require careful observation of the remnant stomach to detect synchronous tumor during follow-up endoscopy.

The known risk factors for LN and distant metastasis are tumor size, tumor differentiation, depth of tumor invasion, and lymphatic and venous invasion [3, 17, 18]. In this study, lymphatic and venous invasion were risk factors for regional LN metastasis in patients with pathological findings beyond the expanded criteria. Furthermore, using the prognostic model, we found that lymphatic and venous invasion, separately and combined, increased the risk of regional LN metastasis compared with no lymphovascular invasion, respectively, in beyond the expanded criteria.

Our study has some limitations. First, it was a retrospective study at a single major referral hospital in Korea. Second, there could have been a selection bias in terms of a difference in tumor characteristics between the observation and surgical groups. Lymphatic and venous invasion as risk factors for regional LN metastasis were more common in the surgery group than in the observation group. There were no significant differences in regional LN metastasis, distant metastasis, and gastric cancer-related death between the two groups because the patients with lower risk factors tended to be more observed than patients with higher risk factors. Long-term clinical outcomes between the groups may vary if the groups were randomized irrespective of risk factors.

Our study also has some strengths. To our knowledge, this is the first study to compare clinical outcomes, including tumor recurrence, according to additional treatment (observation vs. surgery) in patients, with pathological findings beyond the expanded criteria of ESD for EGC, regardless of en bloc status, surgical margin, and lymphatic and vascular invasion, rather than non-curative ESD for EGC. Second, this study investigated the risk factors for active surgery for tumors beyond the expanded criteria and the risk factors for regional LN metastasis using a prognostic model. Finally, this study had a larger number of patients than previous studies.

## Conclusions

In patients with pathological findings beyond the expanded criteria of ESD for EGC with lymphatic and venous invasion, additional surgery should be considered because of the risk for regional LN metastasis. Nevertheless, the clinical course of beyond the expanded criteria of endoscopic submucosal dissection for early gastric cancer showed good prognosis over 98% in 5-year disease specific survival. If additional surgery cannot be performed, a close follow-up with endoscopy and abdominal computed tomography can be considered as an alternative for carefully selected patients without lymphatic and vascular invasion.
